# Impaired energy metabolism of senescent muscle satellite cells is associated with oxidative modifications of glycolytic enzymes

**DOI:** 10.18632/aging.101126

**Published:** 2016-12-04

**Authors:** Martín A. Baraibar, Janek Hyzewicz, Adelina Rogowska-Wrzesinska, Anne-Laure Bulteau, Carina Prip-Buus, Gillian Butler-Browne, Bertrand Friguet

**Affiliations:** ^1^ Sorbonne Universités, UPMC Univ Paris 06, UMR 8256, Biological Adaptation and Ageing- IBPS, CNRS UMR 8256, INSERM U1164, Paris, France; ^2^ Department of Biochemistry and Molecular Biology, University of Southern Denmark, Odense, Denmark; ^3^ Université Paris Descartes, Sorbonne Paris Cité, Faculté de Médecine INSERM U1016, CNRS UMR 8104, Institut Cochin, Paris, France; ^4^ Institut de Myologie, UPMC Univ Paris 06, UMRS INSERM U974, CNRS UMR 7215, CHU Pitié-Salpétrière, Sorbonne Universités, Paris, France

**Keywords:** protein oxidation, proteostasis, myoblasts, aging, cellular senescence, energy metabolism

## Abstract

Accumulation of oxidized proteins is a hallmark of cellular and organismal aging. Adult muscle stem cell (or satellite cell) replication and differentiation is compromised with age contributing to sarcopenia. However, the molecular events related to satellite cell dysfunction during aging are not completely understood. In the present study we have addressed the potential impact of oxidatively modified proteins on the altered metabolism of senescent human satellite cells. By using a modified proteomics analysis we have found that proteins involved in protein quality control and glycolytic enzymes are the main targets of oxidation (carbonylation) and modification with advanced glycation/lipid peroxidation end products during the replicative senescence of satellite cells. Inactivation of the proteasome appeared to be a likely contributor to the accumulation of such damaged proteins. Metabolic and functional analyses revealed an impaired glucose metabolism in senescent cells. A metabolic shift leading to increased mobilization of non-carbohydrate substrates such as branched chain amino acids or long chain fatty acids was observed. Increased levels of acyl-carnitines indicated an increased turnover of storage and membrane lipids for energy production. Taken together, these results support a link between oxidative protein modifications and the altered cellular metabolism associated with the senescent phenotype of human myoblasts.

## INTRODUCTION

Sarcopenia, the age-related loss of muscle mass and force, is the major cause of frailty and fall-related injuries in the elderly [1]. Muscle repair and maintenance is facilitated by resident undifferentiated quiescent mononucleated cells, located beneath the basal lamina with properties of stem cells [2], these are also referred to as to satellite cells [3]. In response to muscle damage, satellite cells are activated to proliferate as myoblasts and migrate to the damaged region of the fiber, where they differentiate and fuse to form myotubes via a similar process to that of myogenesis [4]. This capacity is reduced in the elderly, where satellite cells are unable to efficiently execute the complete repair process, contributing to the loss of muscle mass [5,6].

Although local and systemic changes of the stem cell niche modulate in part the stem cell fate, cell autonomous changes occur as organisms age [7], resulting in the inability of stem cells to proliferate, self-renew or differentiate [8]. In skeletal muscle, previous studies have suggested that increased replicative history rather than chronological aging is the key aspect compromising satellite cell function [9]. Moreover, a hostile local environment in the aging organism may induce premature senescence or alter the stem cell niche leading to a decline in the satellite cell population, which in turn results in reduced growth potential of skeletal muscle *in vivo* [10]. The occurrence of premature senescence of satellite cells in the pathogenesis of muscular dystrophies is also an actual topic of research [11,12]. More importantly, senescent satellite cells show a decreased ability of differentiation and self-renewal [13]. Autophagy has recently been recognized as essential to maintain the mouse muscle stem cells in a quiescent state. Moreover, in aged satellite cells autophagy is impaired and was found to cause premature entry into senescence by increased oxidative stress and loss of proteostasis [14]. However, the molecular mechanisms underlying the dysfunction of senescent myoblasts and how this could participate to muscle loss are not yet completely understood.

Dysregulation of protein homeostasis and accumulation of oxidatively-damaged proteins by reactive oxygen species (ROS) and different processes related to the formation of advanced glycation/lipid peroxidation end products (AGEs and ALEs) are hallmarks of the aging process in different organs and tissues across different species [15,16]. In addition, cellular protein main-tenance systems, such as the proteasome system, are often themselves affected during aging and upon oxidative stress, losing effectiveness over time [17]. Thus, it has been proposed that the accumulation of altered proteins during aging is due to both an increased production of ROS and other toxic compounds, as well as a decreased efficacy of the mechanisms responsible for their removal or repair [18,19].

Among the many types of oxidative protein modifications described, carbonylation is one of the most prominent. Protein carbonylation is irreversible and is related to loss of function or gain of toxic function of the targeted proteins [20]. However, the question, whether protein carbonylation is causally involved in aging and age-related diseases, remains unanswered. In recent years, different studies have evidenced that the “Oxi-proteome” (the build-up of carbonylated proteins) during aging and age-related diseases is composed only by a limited group of proteins [21], indicating that not all proteins have the same propensity for accumulation as oxidatively damaged proteins [22]. This sub-set of oxidation-prone proteins includes those involved in key cellular functions, such as protein quality control and cellular metabolism [22].

Although impairment of protein homeostasis and dysregulation of cellular metabolism have both been described to occur during cellular aging [23-25], up to now, these processes have been viewed as independent events. In this study, we have shown, by integrating modification proteomics and metabolic approaches, a functional connection between oxidative protein modifications and impairment of the related cellular metabolic pathways in senescent human satellite cells.

## RESULTS

### Alteration of protein homeostasis during replicative senescence of human satellite cells

**Figure 1:**
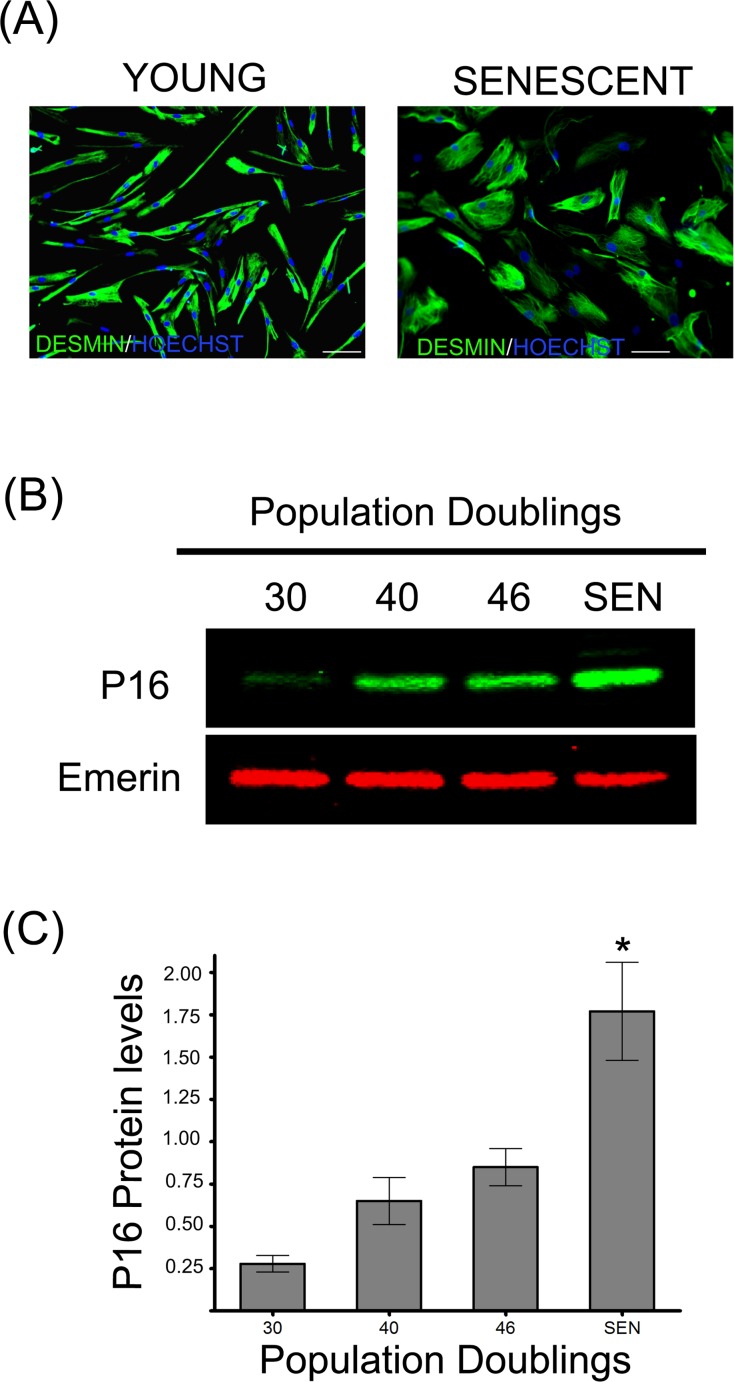
Replicative senescence of human satellite cells *in vitro* (**A**) Immunocytochemistry against desmin evidences morphological changes in senescent human myoblasts (SEN) when compared to young cells (CPD 30). Note the increased diameter and irregular shapes of the formers, as previously described [13]. (**B**) p16 protein levels during replicative senescence in human myoblasts. (**C**) Densitometric analysis of the p16/emerin ratio showed a significant increase (n=3; *P<0.001) in p16 levels during replicative senescence. P16 protein levels are expressed as relative values and shown as mean ± S.D.

Muscle derived satellite cells isolated from a 5-day-old infant were cultivated until they reached replicative senescence at about 48 cumulative population doublings (CPD). Muscle derived satellite cells, also referred as myoblasts, were considered as young until 30 CPD and senescent at the end of their replicative life span when they ceased to respond to mitogenic stimuli and no population doublings were observed during a 4 week period. Senescent myoblasts exhibited typical morphological changes characteristic of senescent cells as they became flattened and enlarged when compared to young cells (Figure 1A). To further validate our model of cellular aging, we investigated the expression of the biomarker of senescence p16 (INK4a) protein (Figure 1B) and found it significantly increased during replicative senescence (Figure 1C). The accumulated p16 will bind the cyclin-dependent kinase 4 (Cdk4), thus inhibiting its activity and blocking the cell-cycle progression [26].

**Figure 2:**
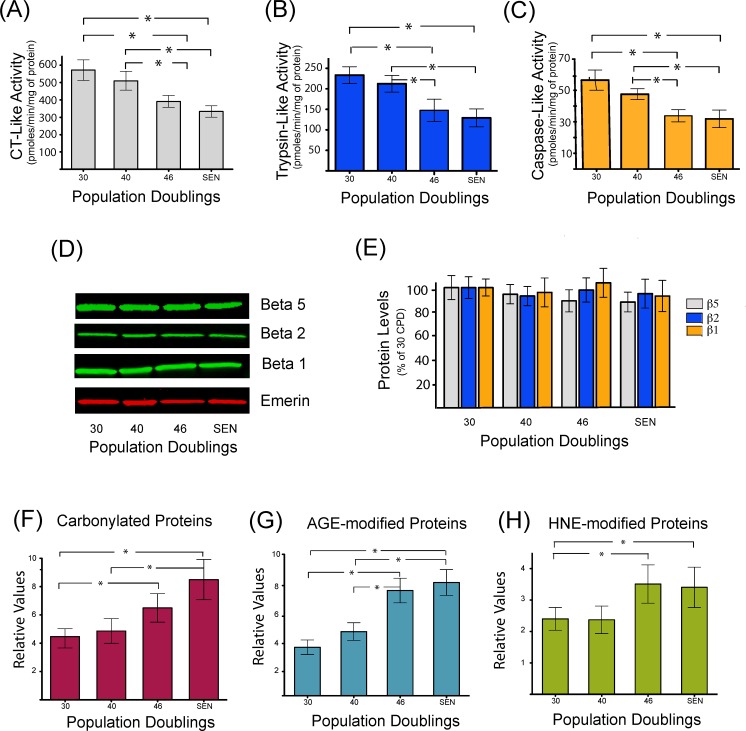
Decreased proteasome activity is associated with the accumulation of oxidized and damaged proteins during replicative senescence Chymotrypsin like (**A**), trypsin-like (**B**), and caspase-like (**C**) peptidase activities of the proteasome were measured during replicative senescence. Protein levels of proteasome catalytic subunits (β1, β2, and β5) were assessed by western blot (**D**) and catalytic subunits protein levels were quantified by densitometric analysis (**E**). Quantification of carbonylated proteins (**F**), proteins modified by different glycated end products (**G**), or modified by the lipid peroxidation product 4-hydroxynonenal (**H**) during replicative senescence of human myoblasts. Protein modifications are expressed as relative values and shown as mean±S.D. (n=3). Data were analyzed by two-way ANOVA followed by Bonferroni's post hoc test. * P<0.05.

Proteasome inhibition appears to be significantly associated with the onset of *in vitro* cellular senescence in multiple cell types such as BJ [27] and WI38 fibroblasts [28]. However, data on senescent human myoblasts have not yet been reported. As depicted in Figure 2, a significant decrease in chymotrypsin-like (Figure 2A), trypsin-like (Figure 2B) and caspase-like (Figure 2C) proteasome peptidase activities was observed in human myoblasts undergoing replicative senescence (starting after 40 CPD). A decreased expression of the catalytic beta subunits in senescent WI 38 human fibroblasts has also been reported [28], explaining the observed decreased activities. However, in human myoblasts, the protein level of the three beta catalytic subunits (Δ1, Δ2 and Δ5) remained constant during their replicative span (Figure 2D and 2E). These results suggest the presence of internal inhibitors, such as highly oxidized and cross-linked proteins that could explain the decreased proteasome activity observed in senescent myoblasts. Previous studies have also shown an inactivation of the proteasome as a consequence of the oxidation of some of its subunits [29-31].

Therefore, to gain further insights into the mechanisms that may be implicated in the impairment of proteasome activity and to further demonstrate the occurrence of deleterious protein modifications, the accumulation of proteins modified by carbonylation (Figure 2F), glycation (Figure 2G) and conjugation with the lipid peroxidation product HNE (Figure 2H), were monitored. Both carbonylated, glycated and HNE-modified proteins were found to be increased during replicative senescence of human myoblasts. Interestingly, the threshold of accumulation starts after 40 CPD, which is coincident with the kinetics of proteasome inhibition.

### Senescence-associated protein modifications target only a subset of the total proteome involved in key cellular pathways

**Figure 3:**
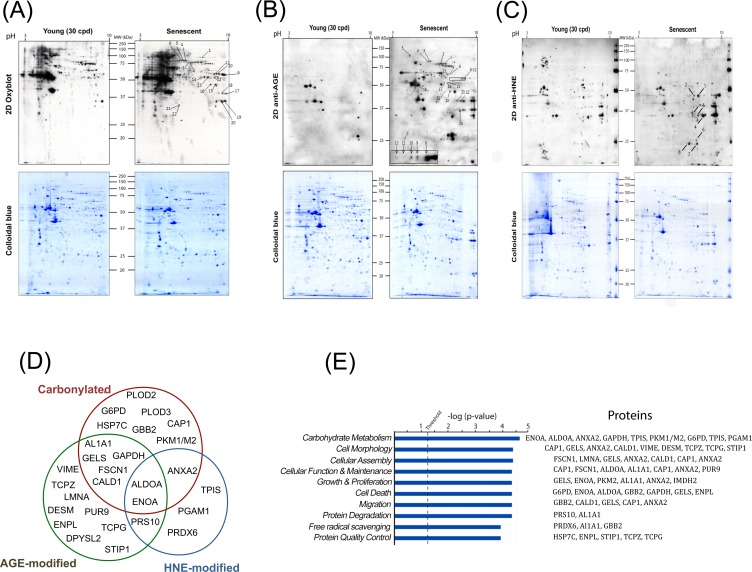
Identification and data mining of modified proteins Cellular protein extracts from young (30 CPD) and senescent human myoblasts were separated by two-dimensional gel electrophoresis. After the second dimension, gels were either stained with colloidal Coomassie Brilliant Blue G (bottom panels) or electrotransferred onto nitrocellulose membranes for subsequently detection of: carbonylated proteins using the OxyBlot^TM^ kit (**A**); glycoxidation protein adducts (**B**) and HNE-modified proteins (**C**). Presented results are from one representative experiment of three independent experiments using three different batches of cells. Numbers refer to the spots evidenced as consistently increased in senescent cells identified by MS/MS. (**D**) Venn diagram depicting the distribution of proteins in relation with the modifications studied (see also Table S1, Table S2 and Table S3). (**E**) Modified proteins were grouped into functional categories through the use of Ingenuity Pathways Analysis. The bars represent the biological functions identified, named in the x-axis. The dotted line represents the threshold above which there are statistically significantly more proteins in a biological function than expected by chance. The identified proteins associated with each pathway are indicated.

Although an increased level of damaged proteins has been shown to accumulate during aging and cellular senescence as well as in certain pathological conditions, in most cases the specific proteins targeted by these modifications have not yet been identified. The identification of these proteins may give insights into the mechanisms by which these damaged proteins could affect cellular functions. For this purpose, total protein extracts from young (30 CPD) and senescent myoblasts were resolved by two-dimensional electrophoresis (2D-PAGE). For each sample, two gel series were prepared in parallel, one for Coomassie colloidal blue staining to visualize the total protein profile and equal loading control, and the second for immunodetection of carbonylated, glycated or HNE-modified proteins (Figure 3A, 3B and 3C). Interestingly, the pattern of the modified proteins was not superimposable with the pattern obtained for the total protein staining, suggesting that certain proteins represent preferential targets for these detrimental modifications.

**Figure 4:**
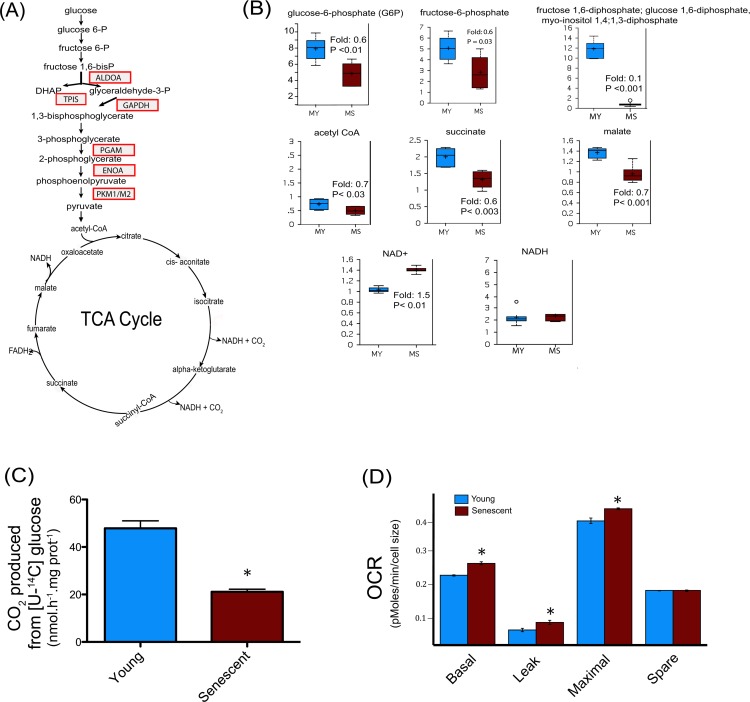
Central metabolism alterations in senescent satellite cells (**A**) Modified enzymes identified in senescent cells and related to the central metabolism are represented in boxes. (**B**) Altered metabolites of central metabolism profiling in young (30 CPD) (MY) and senescent myoblast (MS). For the box plots, the top and bottom of the boxes represent the 75^th^ and 25^th^ percentile, respectively. The solid bar across the box represents the median value, while the + is the mean. Any statistical outliers are represented by a circle. The *Y* axis is the median scaled value (relative level). The fold change and the corresponding p value in senescent cells relative to their young counterpart is indicated in each plot (see also Data set 1). (**C**) Glucose flux in young and senescent myoblasts measured by [U-^14^C] glucose oxidation into ^14^CO_2_. (**D**) Oxygen consumption rates (OCR) of young and senescent myoblasts were monitored using the Seahorse Bioscience Extra Cellular Flux Analyzer. Mitochondrial respiration was determined in basal conditions (growth media), in the presence of oligomycin (leak), and finally in the presence of increasing amounts of carbonyl cyanide m-chlorophenylhydrazone (CCCP; 1-30 μM) to determine the maximal respiration rate. The respiration reserve capacity (spare) was calculated by subtracting the basal to the maximal respiration. The OCR values were normalized to cellular size.

After analyses, during replicative senescence 22 protein spots were shown to be increasingly carbonylated (Figure 3A), 24 were increasingly glycated (Figure 3B), and 8 were increasingly modified by HNE (Figure 3C). Each spot was excised from the gel and analyzed by MALDI-TOF/TOF-MS for protein identification. All spots were successfully identified (Tables S1, S2 and S3). Proteins were identified as targets of one, two or the three modifications (Figure 3D). The identified proteins were then analyzed and grouped by metabolic pathways and cellular functions (Figure 3E). Major biological functions include carbohydrate metabolism, cellular morphology, migration and proliferation, as well as protein quality control, protein degradation and free radical scavenging. We identified 6 enzymes involved in glycolysis to be increasingly modified in the senescent myoblasts (Figure 4A), these were aldolase (ALDOA), triosephosphate isomerase (TPIS), glyceraldehyde 3-phosphate dehydro-genase (GAPDH), phosphoglycerate mutase (PGAM), enolase (ENOA) and pyruvate kinase (PKM1/M2). To further investigate whether these modifications result in a modification of the glycolytic pathway, the steady-state levels of related metabolites were measured and glucose oxidation flux analyses were performed.

### Impairment of glucose oxidation in senescent human myoblasts

Glycolytic intermediates such as glucose-6-phosphate, fructose-6-phosphate, and the isobar containing fructose 1,6-diphosphate, glucose 1,6-diphosphate, and myo-inositol 1,4 or 1,3-diphosphate were all found to be decreased (Figure 4B), suggesting a decreased glucose metabolism via glycolysis in senescent myoblasts. Pyruvate produced through glycolysis is transported into mitochondria to generate acetyl-CoA. The major metabolic fate of acetyl-CoA is to enter the tricarboxylic (TCA) cycle to drive the production of reducing equivalents that in turn fuel the mitochondrial electron transport chain for ATP generation by oxidative phosphorylation. Senescent myoblasts had decreased levels of acetyl-CoA, succinate and malate (Figure 4B), suggesting an altered TCA cycle flux in aged myoblasts. Metabolic failure in senescent myoblasts is also supported by a decreased cellular reducing potential indicated by the increase in the NAD^+^/NADH ratio due to higher NAD^+^ levels whereas NADH remained unchanged (Figure 4B). The oxidative modifications of glycolytic enzymes concomitant with the alteration of metabolites involved in this pathway suggested that myoblasts undergoing replicative senescence present dynamic changes in overall glucose metabolism. Nonetheless, the metabolite profiling analyses measure steady-state levels and are not conclusive in determining the actual flux of glycolytic metabolites. To directly address the hypothesis of impaired glucose metabolism during senescence, we measured [U-^14^C] glucose oxidation into CO_2_, which requires both glycolysis and TCA cycle. As depicted in Figure 4C, ^14^CO_2_ production from [U-^14^C] glucose was decreased by about 40% in senescent myoblasts when compared to their young counterparts. Thus, senescent myoblasts exhibited a substantial decrease in glucose oxidation likely due to impaired glycolysis and TCA cycle.

Since TCA cycle flux depends on the oxidation of reduced equivalents (NADH, FADH_2_) by the mitochondrial respiratory chain, the oxygen consumption rate (OCR) of young and senescent cells was measured by a pharmacological profiling approach using the Seahorse instrument as previously described [32]. Previous studies have shown that the mitochondrial content increases in senescent cells as a function of cell size whereas mitochondrial density remained similar [33,34]. Due to the low number of cells required for the OCR experiments, it was more straightforward to determine cellular size rather than mitochondrial protein content, for which a much higher number of cells is needed. When the OCR were normalized to cellular size, the basal and maximal respiration rates were significantly higher in senescent myoblasts compared to young cells (CPD 30), whereas the spare respiratory capacity was unchanged (Figure 4D). The non-ATP generating respiration (leak) was increased (P<0.01), thus maintaining similar respiratory control in young (1.70 ± 0.2) and senescent cells (1.59 ± 0.1, P = ns). No evidence of uncoupling was observed. Taken together these results indicate that senescent myoblasts have a decreased capacity to oxidize glucose but their mitochondrial respiration capacity is not compromised, suggesting a direct impairment upstream in glycolysis and/or in the TCA cycle as supported by the targeted proteomics and the metabolic analyses.

### Increased mobilization of non-carbohydrate substrates for energy production in senescent human myoblasts

**Figure 5:**
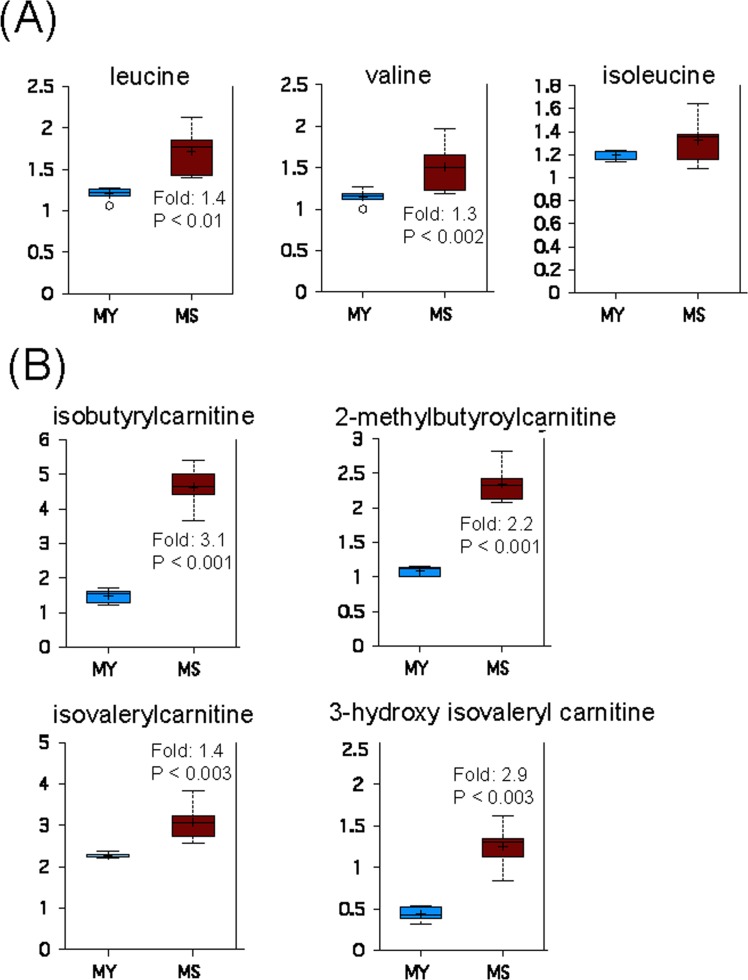
Catabolism of BCAAs is increased during replicative senescence (**A**) Relative levels of branched chain amino acids in young (MY) and senescent (MS) myoblasts. (**B**) Carnitine conjugates of BCAA-derived biochemicals. For details of box plots see Figure legend 4.

Degradation of branched chain amino acids (BCAAs), namely leucine, isoleucine and valine, produces acetyl-CoA, propionyl-CoA, and succinyl-CoA, which can contribute to energy production via the TCA cycle [35]. Increased levels of leucine and valine in senescent myo-blasts (Figure 5A), concomitant with the accumulation of their carnitine conjugates (Figure 5B), suggests an increa-sed catabolism of BCAAs during replicative senescence.

**Figure 6:**
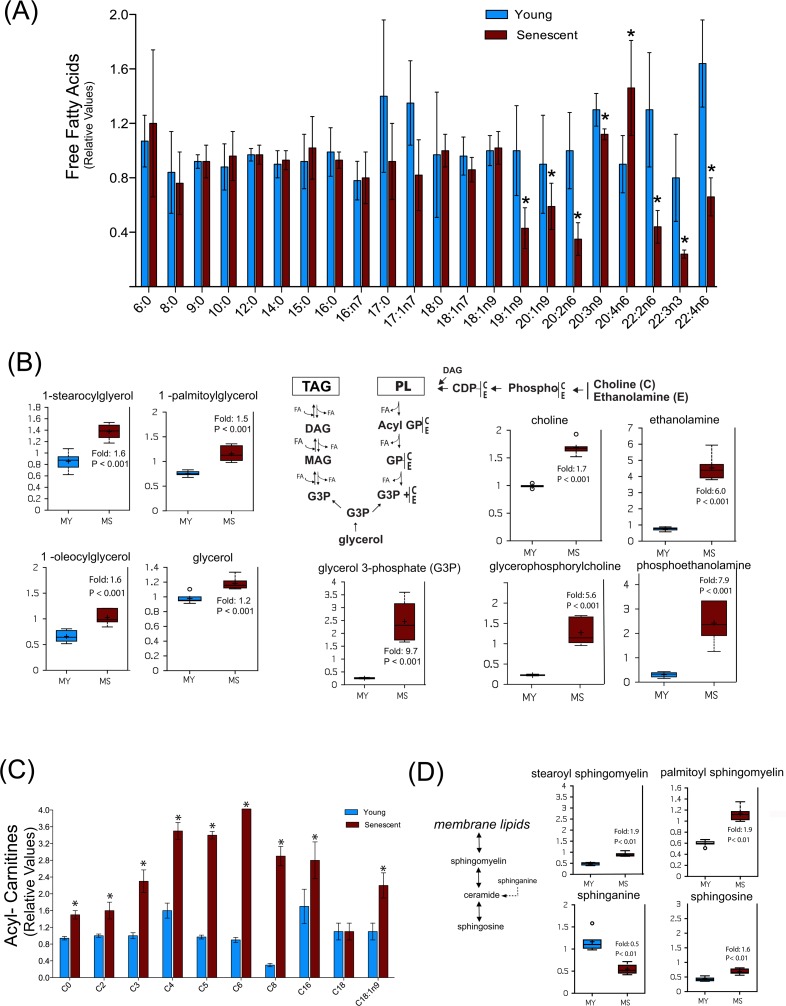
Senescent satellite cells exhibit altered lipid metabolism (**A**) Free fatty acids profiling in young (MY) and senescent myoblasts (MS). (**B**) Increased glycerolipids turnover in senescent cells. PL: phospholipids; TAG: triacylglycerols; DAG: diacylglycerols; MAG: monoacylglycerols; FA: fatty acids; GP: glycerol phosphate. C: choline; E: ethanolamine. (**C**) Free carnitine and acylcarnitine profiling. The acyl chain length (c) is denoted by the corresponding metabolite number (e.g., C0 = free carnitine, C2 = acetylcarnitine; C3 =proprionylcarnitine). (**D**) Sphingolipids metabolism in young and senescent myoblasts. Data are expressed as mean ± S.E.M of six independent experiments. * p <0.05. For details of box plots see Figure legend 4.

Another specific metabolic feature of senescent myoblasts concerns lipid metabolism. Indeed, except for arachidonate (C20:4n6) whose level was increased, several free mono- and polyunsaturated fatty acids with a chain length higher than 18 carbons were found to be decreased, while free medium-chain fatty acids remained unchanged (Figure 6A). This may reflect a decreased synthesis of these specific fatty acids or an increased incorporation into specific cellular lipids. However, increased levels of several monoacyl glycerols and glycerol-3-phosphate (Figure 6B, left panel) as well as of glycerophosphorylcholine, phospho-ethanolamine, choline and ethanolamine (Figure 6B, right panel) in senescent myoblasts suggest an increased turnover of storage lipids (triacyglycerols) and of membrane lipids (glycerophospholipids), pointing towards their mobilization for energy production.

This hypothesis was further reinforced by the observation that the level of several acyl-carnitines was markedly increased during replicative senescence (Figure 6C), suggesting an increased fatty acid β-oxidation. Such changes in lipid metabolism may also reflect changes in membrane composition during replicative senescence, which could also impact on lipid signaling as shown by the increased level of arachidonate (Figure 6A), a precursor of various eicosanoids. Remarkably, we also noticed an enhanced sphingomyelin metabolism in senescent myoblasts as shown by the increased levels of palmitoyl and stearoyl sphingomyelin and shingosine, whereas the sphinganine level decreased (Figure 6D). More importantly, sphin-gomyelin can release ceramide, a signaling molecule implicated in aging [36]. Previous studies have shown that exogenous ceramide inhibited the growth of young dermal fibroblasts and endothelial cells inducing premature senescence by its ability to inhibit DNA synthesis and mitogenesis [37,38]. In addition, ceramide inhibitors increased the expression of myogenic differentiation markers and cell fusion rate in myoblasts, whereas short-chain ceramides inhibited these responses [39]. Indeed, the changes in sphingo-myelinase and ceramide appear to specifically distinguish senescence from quiescent growth arrest. Although we were not able to detect ceramide, increased levels of its degradation product sphingosine and a decrease in its precursor sphinganine suggests that ceramide levels are differentially regulated in senescent myoblasts as compared to young myoblasts.

## DISCUSSION

The occurrence of increased molecular damage due to oxidation is well documented during aging and is believed to have a causative role in cellular aging [22]. Pioneer studies have shown an increase in carbonylated proteins as well as proteins modified with HNE and AGEs in senescent WI-38 human embryonic fibroblasts [40]. It should be noted that the restricted sub-set of modified proteins in senescent myoblasts is different to those identified in senescent fibroblasts, even though similar proteomic approaches were used. Whereas an important number of mitochondrial proteins were found to be increasingly modified in senescent fibroblasts [40], this was not the case for myoblasts, were most of the modified proteins are cytosolic. Our findings do not contradict the molecular damage theory of aging since increased oxidatively damaged proteins were still found in senescent cells. However, these data do challenge the preconception that the increased molecular damage is mainly caused by mitochondrial dysfunction.

Differentially expressed proteins have been identified during replicative senescence of fibroblasts from different organisms [41,42]. However, due to a much smaller data set and fewer proteins identified, they were primarily focused on the differential expression levels of single proteins. Our study is the first large scale application of functional proteomics to replicative senescence of human myoblasts. Along with the comprehensive modification proteomics and metabolic data, coupled with functional analyses it provides a systems-level view to improve our understanding of cellular senescence. We have previously suggested that oxidative damage to key metabolic enzymes could be an important factor contributing to senescence and aging [21]. This study gives credit to our hypothesis and brings mechanistic insights as to how metabolic dysregulation during cellular aging may result, at least in part, from the oxidative damage of a restricted set of proteins. Indeed, the oxidative modification of enzymes involved in the glycolytic pathway is concomitant with a decreased cellular reducing potential, as demonstrated by the increased NAD^+^/NADH ratio, and decreased glucose oxidation. In addition, we have demonstrated that the functionality of the mitochondrial respiratory chain is not affected in human myoblasts during replicative senescence, indicating that the metabolic shift which we observed is most likely due to an impairment in glycolysis and/or TCA cycle. Oxidative modifications of enzymes involved in glycolysis and the TCA cycle have also been suggested to be an important pathophysiological factor in age-related diseases, such as neurodegenerative diseases [43]. Other studies have shown that the inhibition of glycolytic enzymes, such as GAPDH and PGAM by siRNA can induce premature senescence [44]. In contrast, the proliferative capacity of murine embryonic stem cells closely correlates with high activity of different glycolytic enzymes, elevated glycolytic flux, and low mitochondrial oxygen consumption [45].

Previous studies in primary culture of human myoblasts have shown that a single treatment with exogenous hydrogen peroxide resulted in the loss of viability, a shorter population-doubling competence, and a significantly decreased proliferative capacity, suggesting an oxidative stress-induced premature senescence effect [5]. The proteome changes of adult human muscle stem cells in response to oxidative stress have been characterized [46]. Data mining of the identified carbonylated proteins suggested that carbohydrate metabolism is also affected by oxidative stress and that the impairment of these pathways may be implicated in the oxidative stress-induced cellular dysfunction. Our findings suggest an overall link between the bioenergetic status of myoblasts and their proliferation capacity. The metabolic changes we have identified during senescence have been shown to play a role in aging at the organismal level [47,48]. Senescent myoblasts exhibited a metabolic shift, with a pronounc-ed down regulation in glycolysis coupled with an increased oxidation of long chain fatty acids and branched chain amino acids. In addition, changes in lipid metabolism in senescent myoblasts may reflect membrane remodeling and changes in lipid-derived signaling molecules.

In summary, we have described the occurrence of increased and targeted protein oxidative damage together with impaired cellular metabolism during replicative senescence of adult human muscle stem cells. By combining modification proteomics and metabolic analyses, in addition to functional studies, we have identified cellular pathways that are implicated in such processes. This study establishes a new link between oxidative protein modifications and the altered cellular metabolism associated with the senescent phenotype. Future studies should provide a comprehensive view of benefits to be gained from increased protection against oxidative modification of such specific proteins and/or the stimulation of their elimination.

## METHODS

### Primary culture of human muscle satellite cells

Satellite cells were isolated as previously described in accordance with the French legislation on ethical rules [49]. Using the explants method, upon autopsy, neonatal satellite cells were isolated from the quadriceps muscle of a 5-day-old female infant that died due to a developmental heart defect without showing signs of neuromuscular disorder. Cells were expanded in growth medium (DMEM 65%(v/v), 199 media 15% (v/v) and fetal bovine serum 20% (v/v)). Cultures were performed in a humidified atmosphere with 5% CO_2_ at 37°C until sub-confluence. Magnetic-activated cell sorting (MACS) was used to purify myogenic cells using Mini MACS (Miltenyi Biotec, Ber-gisch Gladbach, Germany) using anti-neural cell adhesion molecule (NCAM) antibodies, according to manufacturer instructions. Sub-confluent cultures were obtained by seeding 4×10^5^cells/225 cm^2^ culture flasks until they entered senescence. For each passage, cumulative population doublings (CPD) was calculated as: log (N/n)/ln 2, where N is the number of cells counted and n is the number of cells initially plated. In all experiments, myogenicity was greater than 95% as assessed by desmin immunostaining after 24h of differentiation by serum reduction to 2% (v/v).

### Proteasome peptidase activities

Cell pellets derived from three independent experiments of cells at CPD: 30, 40, 46 and senescent were homogenized in an extraction buffer containing 50 mM Tris-HCl (pH 7) and 1 mM dithiothreitol. After clearance by centrifugation, protein concentration was determined by the Bradford method (Bio-Rad protein assay). Peptidase activities were assayed using the fluorescent peptide substrates: succinyl-Leu-Leu-Val-Tyr-aminomethylcoumarin (LLVY-AMC) for the chymotrypsin-like activity, Ac-Arg-Leu-Arg-amino-methylcoumarin (RLR-AMC) for the trypsin-like activity and N-benzyloxycarbonyl-Leu-Leu-Glu-Δ-naphthylamide (LLE-NA) for the peptidyl-glutamyl peptide hydrolyzing or caspase-like activity, as previously described [46]. Proteasome peptidase activities were determined as the difference between total activity and the remaining activity of the crude lysates in the presence of the proteasome inhibitor MG132 (20 μM). All measurements were performed in three different batches of cells, and results were expressed as mean ± standard deviation (SD). Data were analyzed two-way ANOVA followed by Bonferroni's post hoc test. * P<0.01.

### Protein extraction for proteomics analyses

Cellular pellets derived from three independent experiments were homogenized using a lysis buffer (10 mM tris-HCl (pH 7.4), 8 M urea, 2 M thiourea, 4% (w/v) CHAPS) and clarified by centrifugation. Proteins in the supernatant were precipitated using the 2-D Clean-Up kit (GE Healthcare). Protein precipitates were then resuspended in lysis buffer and protein quantifica-tion was performed as described above.

### Immunodetection of carbonylated, HNE- and AGE-modified proteins after 1D or 2D gel electrophoresis

Immunoblot experiments for carbonyl detection were performed using the OxyBlot^TM^ kit, according to the manufacturer instructions. Detection of HNE-modified proteins was achieved after overnight incubation at 4°C with polyclonal antibodies raised against HNE-modified KLH (ab46544, Abcam). For AGE-modified proteins, membranes were incubated with polyclonal antibodies raised against AGE-modified RNAse [50]. After washing, secondary antibodies IRDye were used, and membranes were scanned and quantified by the ODYSSEY infrared imaging system (LI-COR, Les Ulis, France). Two-dimensional polyacrylamide gel electro-phoresis (2D PAGE) separation of proteins was performed as described previously [46]. For each sample, two gels were performed in parallel, one for colloidal blue staining of total proteins and other for electro-blotting onto nitrocellulose membranes. Membranes were then incubated for 2h in the blocking solution and immunodetection was performed as described above. Membranes were revealed using Amersham ECL Plus Western Blotting Detection System. Films were digitized with UMAX UTA-100 scanner (GE Healthcare, Saclay, France) and densitometry analyses were performed using NIH ImageJ or Image Master 2D Platinum 7 software (GE Healthcare).

### Proteomics data acquisition and analysis

Spot detection and quantification was carried out using the Image Master 2D Platinum 7 software (GE Healthcare). For each spot identified by Coomassie colloidal blue as well as from the corresponding immunodetection (carbonylated, AGE- and HNE-modified proteins), a percentage volume was obtained in pixels (%Vol). The %Vol corresponds to a normalized value of the spot volume by considering the total volume of all the spots present in the gels or on films. %Vol of carbonylated spots from young (CPD 30) and senescent cells were normalized with %Vol of the corresponding Coomassie-stained spot to obtain a normalized %Vol (N%Vol). The N%Vol of senescent cells was divided by the N%Vol of young cells to obtain the Relative Modification Index ratio (RMI ratio). Spots with an RMI ratio ≥1.3 were considered as increasingly modified in the senescent cells. All measurements were repeated a minimum of three times and results are expressed as mean ± standard deviation (S.D.). Data were tested for normality and statistical significance for the comparison of two groups using student's t-test: *P < 0.01.

### In-gel digestion, mass spectrometry protein identification and databases searches

Spots of interest were manually excised from gels and proteins were digested with trypsin as described previously [40]. Peptide mass spectra were acquired in positive reflector mode on a 4800 Plus MALDI TOF/TOF™ Analyzer (Applied Biosystems, Foster City, CA, USA) using 20 kV of acceleration voltage. MS and MS/MS peak lists were combined into search files and used to search SwissProt database using the Mascot search engine (Matrix Science Ltd, London, UK). Mascot protein scores greater than 56 are significant at p<0.05.

### Metabolic analyses

Metabolites profiling analysis was performed by Metabolon, Inc. as previously described [51]. Cellular pellets (10^6^ cells) from six different batches from young (CPD30) and senescent myoblasts were accessioned into the Metabolon LIMS system.

#### Ultrahigh performance liquid chromatography/Mass Spectroscopy (UPLC/MS/MS)

The LC/MS portion of the platform was based on a Waters ACQUITY ultra-performance liquid chroma-tography (UPLC) and a Thermo-Finnigan linear trap quadrupole (LTQ) mass spectrometer. Sample extracts were dried and then reconstituted in acidic or basic LC-compatible solvents, each of which contained 8 or more injection standards at fixed concentrations to ensure injection and chromatographic consistency.

#### Gas chromatography/Mass Spectroscopy (GC/MS)

The samples destined for GC/MS analysis were re-dried under vacuum desiccation for a minimum of 24h prior to being derivatized under dried nitrogen using bistrimethylsilyl-triflouroacetamide (BSTFA). Samples were analyzed on a Thermo-Finnigan Trace DSQ fast-scanning single-quadrupole mass spectrometer using electron impact ionization.

#### Data extraction and compound identification

Raw data was extracted, peak-identified and QC processed using Metabolon's hardware and software [52]. Welch's two-sample *t*-test was used to identify biochemicals that differed significantly between experimental groups.

### Measurement of glucose flux and oxygen consumption rates

During the last 24 h of culture, the growth medium from young and senescent myoblasts was replaced by a medium containing 5 mM [U-^14^C]D-glucose (133 μCi/nmol) and insulin (10 nM). After 24h, the medium was then collected to determine ^14^CO_2_, whereas cells were washed three times with ice-cold PBS and scraped for protein quantification. Oxygen consumption rate (OCR) was determined using Seahorse XF-96 extracellular Flux analyzer (Seahorse Bioscience, Billerica, MA, USA) as previously described [32]. Briefly, young (CPD 30) and senescent cells were seeded in the 96-well XF-96 cell in sixteen replicates at 20.000 cells/well in growth medium. Thirty minutes prior to the run, cells were equilibrated with new culture medium in a 37°C CO_2_ incubator for pH and tem-perature stabilization. Three mitochondrial inhibitors: oligomycin (1μM), carbonyl cyanide m-chloro-phenylhydrazone (CCCP; 1-30 μM) and KCN (1mM) were sequentially injected (protocol, 12 min of equilibration, followed by measurements of 3 min, separated by mixing for 3 min). After all the measure-ments were completed, cells were dissociated and cell number was measured using a BD accuri cytometer (BD, Le Pont –de – Claix, France) while cell size was assessed using flow cytometry by monitoring light scattering.

## SUPPLEMENTARY MATERIAL AND TABLE











## Abbreviations

AGEs: advanced glycation end products
ALEs: advanced lipid peroxidation end products
BCAAs: branched chain aminoacids
CCCP: carbonyl cyanide m-chlorophenylhydrazone
CPD: cumulative population doublings
HNE: 4-hydroxynonenal
MS: mass spectrometry
OCR: oxygen consumption rate

## References

[1] Cruz-Jentoft AJ, Baeyens JP, Bauer JM, Boirie Y, Cederholm T, Landi F, Martin FC, Michel JP, Rolland Y, Schneider SM, Topinková E, Vandewoude M, Zamboni M (2010). and European Working Group on Sarcopenia in Older People. Sarcopenia: European consensus on definition and diagnosis: Report of the European Working Group on Sarcopenia in Older People. Age Ageing.

[2] Lepper C, Partridge TA, Fan CM (2011). An absolute requirement for Pax7-positive satellite cells in acute injury-induced skeletal muscle regeneration. Development.

[3] Mauro A (1961). Satellite cell of skeletal muscle fibers. J Biophys Biochem Cytol.

[4] Chargé SB, Rudnicki MA (2004). Cellular and molecular regulation of muscle regeneration. Physiol Rev.

[5] Renault V, Thornell LE, Butler-Browne G, Mouly V (2002). Human skeletal muscle satellite cells: aging, oxidative stress and the mitotic clock. Exp Gerontol.

[6] Conboy IM, Rando TA (2005). Aging, stem cells and tissue regeneration: lessons from muscle. Cell Cycle.

[7] Campisi J, Robert L (2014). Cell senescence: role in aging and age-related diseases. Interdiscip Top Gerontol.

[8] Bigot A, Duddy WJ, Ouandaogo ZG, Negroni E, Mariot V, Ghimbovschi S, Harmon B, Wielgosik A, Loiseau C, Devaney J, Dumonceaux J, Butler-Browne G, Mouly V, Duguez S (2015). Age-Associated Methylation Suppresses SPRY1, Leading to a Failure of Re-quiescence and Loss of the Reserve Stem Cell Pool in Elderly Muscle. Cell Reports.

[9] Thornell LE, Lindström M, Renault V, Mouly V, Butler-Browne GS (2003). Satellite cells and training in the elderly. Scand J Med Sci Sports.

[10] Kudryashova E, Kramerova I, Spencer MJ (2012). Satellite cell senescence underlies myopathy in a mouse model of limb-girdle muscular dystrophy 2H. J Clin Invest.

[11] Wallace GQ, McNally EM (2009). Mechanisms of muscle degeneration, regeneration, and repair in the muscular dystrophies. Annu Rev Physiol.

[12] Brack AS, Rando TA (2012). Tissue-specific stem cells: lessons from the skeletal muscle satellite cell. Cell Stem Cell.

[13] Bigot A, Jacquemin V, Debacq-Chainiaux F, Butler-Browne GS, Toussaint O, Furling D, Mouly V (2008). Replicative aging down-regulates the myogenic regulatory factors in human myoblasts. Biol Cell.

[14] García-Prat L, Martínez-Vicente M, Perdiguero E, Ortet L, Rodríguez-Ubreva J, Rebollo E, Ruiz-Bonilla V, Gutarra S, Ballestar E, Serrano AL, Sandri M, Muñoz-Cánoves P (2016). Autophagy maintains stemness by preventing senescence. Nature.

[15] Levine RL, Stadtman ER (2001). Oxidative modification of proteins during aging. Exp Gerontol.

[16] Nyström T (2005). Role of oxidative carbonylation in protein quality control and senescence. EMBO J.

[17] Baraibar MA, Friguet B (2012). Changes of the proteasomal system during the aging process. Prog Mol Biol Transl Sci.

[18] Friguet B (2006). Oxidized protein degradation and repair in ageing and oxidative stress. FEBS Lett.

[19] Chondrogianni N, Petropoulos I, Grimm S, Georgila K, Catalgol B, Friguet B, Grune T, Gonos ES (2014). Protein damage, repair and proteolysis. Mol Aspects Med.

[20] Baraibar MA, Barbeito AG, Muhoberac BB, Vidal R (2012). A mutant light-chain ferritin that causes neuro-degeneration has enhanced propensity toward oxidative damage. Free Radic Biol Med.

[21] Baraibar MA, Friguet B (2013). Oxidative proteome modifications target specific cellular pathways during oxidative stress, cellular senescence and aging. Exp Gerontol.

[22] Baraibar MA, Liu L, Ahmed EK, Friguet B (2012). Protein oxidative damage at the crossroads of cellular senescence, aging, and age-related diseases. Oxid Med Cell Longev.

[23] Morimoto RI, Cuervo AM (2009). Protein homeostasis and aging: taking care of proteins from the cradle to the grave. J Gerontol A Biol Sci Med Sci.

[24] Koga H, Kaushik S, Cuervo AM (2011). Protein homeostasis and aging: the importance of exquisite quality control. Ageing Res Rev.

[25] Cecarini V, Gee J, Fioretti E, Amici M, Angeletti M, Eleuteri AM, Keller JN (2007). Protein oxidation and cellular homeostasis: emphasis on metabolism. Biochim Biophys Acta.

[26] Rayess H, Wang MB, Srivatsan ES (2012). Cellular senescence and tumor suppressor gene p16. Int J Cancer.

[27] Sitte N, Merker K, Von Zglinicki T, Grune T, Davies KJ (2000). Protein oxidation and degradation during cellular senescence of human BJ fibroblasts: part I--effects of proliferative senescence. FASEB J.

[28] Chondrogianni N, Stratford FL, Trougakos IP, Friguet B, Rivett AJ, Gonos ES (2003). Central role of the proteasome in senescence and survival of human fibroblasts: induction of a senescence-like phenotype upon its inhibition and resistance to stress upon its activation. J Biol Chem.

[29] Bulteau AL, Lundberg KC, Humphries KM, Sadek HA, Szweda PA, Friguet B, Szweda LI (2001). Oxidative modification and inactivation of the proteasome during coronary occlusion/reperfusion. J Biol Chem.

[30] Carrard G, Dieu M, Raes M, Toussaint O, Friguet B (2003). Impact of ageing on proteasome structure and function in human lymphocytes. Int J Biochem Cell Biol.

[31] Viteri G, Carrard G, Birlouez-Aragón I, Silva E, Friguet B (2004). Age-dependent protein modifications and declining proteasome activity in the human lens. Arch Biochem Biophys.

[32] Qian W, Van Houten B (2010). Alterations in bioenergetics due to changes in mitochondrial DNA copy number. Methods.

[33] Lee HC, Yin PH, Chi CW, Wei YH (2002). Increase in mitochondrial mass in human fibroblasts under oxidative stress and during replicative cell senescence. J Biomed Sci.

[34] Hutter E, Renner K, Pfister G, Stöckl P, Jansen-Dürr P, Gnaiger E (2004). Senescence-associated changes in respiration and oxidative phosphorylation in primary human fibroblasts. Biochem J.

[35] Newgard CB, An J, Bain JR, Muehlbauer MJ, Stevens RD, Lien LF, Haqq AM, Shah SH, Arlotto M, Slentz CA, Rochon J, Gallup D, Ilkayeva O (2009). A branched-chain amino acid-related metabolic signature that differentiates obese and lean humans and contributes to insulin resistance. Cell Metab.

[36] Cutler RG, Kelly J, Storie K, Pedersen WA, Tammara A, Hatanpaa K, Troncoso JC, Mattson MP (2004). Involvement of oxidative stress-induced abnormalities in ceramide and cholesterol metabolism in brain aging and Alzheimer's disease. Proc Natl Acad Sci USA.

[37] Venable ME, Lee JY, Smyth MJ, Bielawska A, Obeid LM (1995). Role of ceramide in cellular senescence. J Biol Chem.

[38] Venable ME, Yin X (2009). Ceramide induces endothelial cell senescence. Cell Biochem Funct.

[39] Mebarek S, Komati H, Naro F, Zeiller C, Alvisi M, Lagarde M, Prigent AF, Némoz G (2007). Inhibition of de novo ceramide synthesis upregulates phospholipase D and enhances myogenic differentiation. J Cell Sci.

[40] Ahmed EK, Rogowska-Wrzesinska A, Roepstorff P, Bulteau AL, Friguet B (2010). Protein modification and replicative senescence of WI-38 human embryonic fibroblasts. Aging Cell.

[41] Benvenuti S, Cramer R, Quinn CC, Bruce J, Zvelebil M, Corless S, Bond J, Yang A, Hockfield S, Burlingame AL, Waterfield MD, Jat PS (2002). Differential proteome analysis of replicative senescence in rat embryo fibroblasts. Mol Cell Proteomics.

[42] Dierick JF, Kalume DE, Wenders F, Salmon M, Dieu M, Raes M, Roepstorff P, Toussaint O (2002). Identification of 30 protein species involved in replicative senescence and stress-induced premature senescence. FEBS Lett.

[43] Martínez A, Portero-Otin M, Pamplona R, Ferrer I (2010). Protein targets of oxidative damage in human neurodegenerative diseases with abnormal protein aggregates. Brain Pathol.

[44] Kondoh H, Lleonart ME, Gil J, Wang J, Degan P, Peters G, Martinez D, Carnero A, Beach D (2005). Glycolytic enzymes can modulate cellular life span. Cancer Res.

[45] Kondoh H, Lleonart ME, Nakashima Y, Yokode M, Tanaka M, Bernard D, Gil J, Beach D (2007). A high glycolytic flux supports the proliferative potential of murine embryonic stem cells. Antioxid Redox Signal.

[46] Baraibar MA, Hyzewicz J, Rogowska-Wrzesinska A, Ladouce R, Roepstorff P, Mouly V, Friguet B (2011). Oxidative stress-induced proteome alterations target different cellular pathways in human myoblasts. Free Radic Biol Med.

[47] Houtkooper RH, Argmann C, Houten SM, Cantó C, Jeninga EH, Andreux PA, Thomas C, Doenlen R, Schoonjans K, Auwerx J (2011). The metabolic footprint of aging in mice. Sci Rep.

[48] Baker DJ, Wijshake T, Tchkonia T, LeBrasseur NK, Childs BG, van de Sluis B, Kirkland JL, van Deursen JM (2011). Clearance of p16Ink4a-positive senescent cells delays ageing-associated disorders. Nature.

[49] Decary S, Mouly V, Hamida CB, Sautet A, Barbet JP, Butler-Browne GS (1997). Replicative potential and telomere length in human skeletal muscle: implications for satellite cell-mediated gene therapy. Hum Gene Ther.

[50] Verbeke P, Perichon M, Borot-Laloi C, Schaeverbeke J, Bakala H (1997). Accumulation of advanced glycation endproducts in the rat nephron: link with circulating AGEs during aging. J Histochem Cytochem.

[51] Reitman ZJ, Jin G, Karoly ED, Spasojevic I, Yang J, Kinzler KW, He Y, Bigner DD, Vogelstein B, Yan H (2011). Profiling the effects of isocitrate dehydrogenase 1 and 2 mutations on the cellular metabolome. Proc Natl Acad Sci USA.

[52] Dehaven CD, Evans AM, Dai H, Lawton KA (2010). Organization of GC/MS and LC/MS metabolomics data into chemical libraries. J Cheminform.

