# Supplementary material for: Impaired energy metabolism of senescent muscle satellite cells is associated with oxidative modifications of glycolytic enzymes
Source: Aging (Albany NY). 2016 Dec 4;8(12):3375–88. doi: 10.18632/aging.101126 (PMC5270674; doi:10.18632/aging.101126)
Supplement: Supplementary file 1 [file aging-08-3375-s001.pdf]

SUPPLEMENTARY MATERIAL

Please browse links in Full Text version to see Supplementary Material listings:

Table S1. Carbonylated proteins in senescent myoblasts
Table S2. Proteins modified by glycation adducts in senescent myoblasts
Table S3. HNE modified proteins in senescent satellite cells
Data Set 1. Pathway heat map of statistically significant biochemicals profiled in this study.
