# Supplementary material for: Impaired energy metabolism of senescent muscle satellite cells is associated with oxidative modifications of glycolytic enzymes
Source: Aging (Albany NY). 2016 Dec 4;8(12):3375–88. doi: 10.18632/aging.101126 (PMC5270674; doi:10.18632/aging.101126)
Supplement: Supplementary file 2 [file aging-08-3375-s002.docx]

**Supplementary Table 1** (Related to Figure 3A). Carbonylated proteins in senescent myoblasts

| Protein spot no^a^ | Identified protein name | Swiss-Prot accession no^b^ | Mascot score^c^ | Sequence coverage (%)^d^ | No. of matched peptides^e^ | No. of sequenced peptides^f^ | Theoretical protein mass (Da)^g^ | Theoretical PI^h^ | RMI ratio^i^ |
| --- | --- | --- | --- | --- | --- | --- | --- | --- | --- |
| 1 | Procollagen-lysine,2-oxoglutarate 5-dioxygenase 2 | PLOD2 | 263 | 15 | 14 | 5 | 84632 | 6,24 | 1,5 |
| 2 | Caldesmon | CALD1 | 397 | 26 | 42 | 12 | 93194 | 5,63 | 4,69 |
| 3 | Procollagen-lysine,2-oxoglutarate 5-dioxygenase 3 | PLOD3 | 115 | 12 | 13 | 2 | 84731 | 5,69 | 1,4 |
| 4 | Gelsolin | GELS | 882 | 45 | 38 | 11 | 85644 | 5,72 | 1,86 |
| 5 | Gelsolin | GELS | 893 | 36 | 37 | 13 | 85644 | 5,72 | h |
| 6 | Gelsolin | GELS | 394 | 32 | 24 | 5 | 85644 | 5,72 | 3,65 |
| 7 | Heat shock cognate 71 kDa protein | HSP7C | 1060 | 48 | 40 | 14 | 70761 | 5,37 | 1,45 |
| 8 | Heat shock cognate 71 kDa protein | HSP7C | 954 | 46 | 13 | 13 | 70854 | 5,37 | 1,77 |
| 9 | Pyruvate kinase isozymes M1/M2 | PKM1/M2 | 785 | 59 | 42 | 12 | 57900 | 7,96 | 6,66 |
| 10 | Pyruvate kinase isozymes M1/M2 | PKM1/M2 | 208 | 31 | 14 | 4 | 57900 | 7,96 | 2,32 |
| 11 | Fascin | FSCN1 | 116 | 20 | 13 | 3 | 54496 | 6,84 | 1,61 |
| 12 | Adenylyl cyclase-associated protein 1 | CAP1 | 351 | 35 | 19 | 5 | 51823 | 8,27 | 2,76 |
| 13 | Glucose-6-phosphate 1-dehydrogenase | G6PD | 72 | 17 | 16 | 4 | 59219 | 6,39 | 2,75 |
| 14 | Retinal dehydrogenase 1 | AL1A1 | 682 | 32 | 23 | 10 | 54827 | 6,3 | 1,74 |
| 15 | Alpha-enolase | ENOA | 1040 | 47 | 32 | 10 | 47139 | 7,01 | 1,83 |
| 16 | Alpha-enolase | ENOA | 927 | 48 | 34 | 12 | 47139 | 7,01 | 4,16 |
| 17 | Fructose-bisphosphate aldolase A | ALDOA | 596 | 50 | 23 | 8 | 39395 | 8,3 | h |
| 18 | Fructose-bisphosphate aldolase A | ALDOA | 303 | 42 | 18 | 5 | 39395 | 8,3 | 4,88 |
| 19 | Glyceraldehyde-3-phosphate dehydrogenase | GAPDH | 373 | 36 | 15 | 3 | 36030 | 8,58 | h |
| 20 | Annexin A2 | ANXA2 | 1360 | 58 | 39 | 17 | 38580 | 7,56 | 1,88 |
| 21 | Guanine nucleotide-binding protein G(I)/G(S)/G(T) subunit beta-2 | GBB2 | 130 | 15 | 10 | 4 | 37307 | 5,6 | 2,22 |
| 22 | Guanine nucleotide-binding protein G(I)/G(S)/G(T) subunit beta-2 | GBB2 | 80 | 10 | 4 | 1 | 37307 | 5,6 | 1,77 |

Spots of interest were identified by MS as described in experimental procedures. Protein spots no (a) refer to numbered spots on Fig 3A. For each spot, different parameters clarifying protein identification by MS are indicated [accession number (b), mascot score (c), % sequence coverage (d), no. of matched peptides (e), no. of sequenced peptides (f), theoretical protein mass (g) and theoretical PI (h)]. RMI ratio (i) represents the Relative Modification Index Ratio and h means that the RMI ratio is higher than 5.
