# Supplementary material for: Impaired energy metabolism of senescent muscle satellite cells is associated with oxidative modifications of glycolytic enzymes
Source: Aging (Albany NY). 2016 Dec 4;8(12):3375–88. doi: 10.18632/aging.101126 (PMC5270674; doi:10.18632/aging.101126)
Supplement: Supplementary file 3 [file aging-08-3375-s003.docx]

**Supplementary Table 2** (Related to Figure 3B). Proteins modified by glycation adducts in senescent myoblasts

| Protein spot no^a^ | Identified protein name | Swiss-Prot accession no^b^ | Mascot score^c^ | Sequence coverage (%)^d^ | No. of matched peptides^e^ | No. of sequenced peptides^f^ | Theoretical protein mass (Da)^g^ | Theoretical PI^h^ | RMI ratio^i^ |
| --- | --- | --- | --- | --- | --- | --- | --- | --- | --- |
| 1 | Caldesmon | CALD1 | 268 | 20 | 15 | 6 | 93250 | 5.63 | 2,89 |
| 2 | Caldesmon | CALD1 | 179 | 11 | 13 | 4 | 93250 | 5.63 | h |
| 3 | Gelsolin | GELS | 1120 | 37 | 36 | 13 | 85698 | 5.9 | 4,15 |
| 4 | Gelsolin | GELS | 904 | 33 | 34 | 9 | 85698 | 5.9 | 2,11 |
| 5 | Endoplasmin | ENPL | 1460 | 33 | 46 | 24 | 92469 | 4.76 | n |
| 6 | Prelamin-A/C | LMNA | 195 | 9 | 11 | 4 | 74139 | 6.57 | 4,21 |
| 7 | Prelamin-A/C | LMNA | 404 | 16 | 19 | 8 | 74139 | 6.57 | 1,3 |
| 8 | Prelamin-A/C | LMNA | 416 | 21 | 20 | 9 | 74139 | 6.57 | h |
| 9 | Stress-induced-phosphoprotein 1 | STIP1 | 95 | 11 | 11 | 3 | 62639 | 6.4 | h |
| 10 | Bifunctional purine biosynthesis protein PURH | PUR9 | 115 | 9 | 7 | 2 | 64616 | 6.27 | h |
| 11 | Prelamin-A/C | LMNA | 195 | 9 | 9 | 4 | 74139 | 6.57 | 2,34 |
| 12 | T-complex protein 1 subunit zeta | TCPZ | 566 | 31 | 26 | 10 | 58024 | 6.24 | h |
| 13 | Dihydropyrimidinase-related protein 2 | DPYSL2 | 455 | 35 | 24 | 7 | 62294 | 5.95 | 1,65 |
| 14 | T-complex protein 1 subunit gamma | TCPG | 249 | 25 | 18 | 6 | 60534 | 6.1 | 3,94 |
| 15 | Fascin | FSCN1 | 371 | 25 | 17 | 8 | 54530 | 6.84 | h |
| 16 | Retinal dehydrogenase 1 | AL1A1 | 606 | 32 | 20 | 8 | 54862 | 6.3 | h |
| 17 | Retinal dehydrogenase 1 | AL1A1 | 610 | 32 | 22 | 9 | 54862 | 6.3 | h |
| 18 | Desmin | DESM | 906 | 43 | 23 | 8 | 53536 | 5,21 | 1,8 |
| 19 | Vimentin | VIME | 1460 | 57 | 40 | 20 | 53652 | 5.06 | 4,59 |
| 20 | Alpha-enolase | ENOA | 522 | 25 | 23 | 7 | 47169 | 7.01 | 2,53 |
| 21 | Alpha-enolase | ENOA | 917 | 35 | 28 | 12 | 47169 | 7.01 | h |
| 22 | Fructose-bisphosphate aldolase A | ALDOA | 568 | 34 | 19 | 8 | 39420 | 8.3 | h |
| 23 | 26S protease regulatory subunit 10B | PRS10 | 145 | 16 | 8 | 3 | 44173 | 7.09 | 3,41 |
| 24 | Glyceraldehyde-3-phosphate dehydrogenase | GAPDH | 363 | 41 | 17 | 6 | 36053 | 8.57 | 3,18 |

Spots of interest were identified by MS as described in Experimental procedures. Protein spots no (a) refer to numbered spots on Fig 3B. For each spot, different parameters clarifying protein identification by MS are indicated [accession number (b), mascot score (c), % sequence coverage (d), no. of matched peptides (e), no. of sequenced peptides (f), theoretical protein mass (g) and theoretical PI (h)]. RMI ratio (i) represents the Relative Modification Index Ratio. h means that the RMI ratio is higher than 5, n mean that the modified protein was only detected on the senescent samples.
