# Supplementary material for: Impaired energy metabolism of senescent muscle satellite cells is associated with oxidative modifications of glycolytic enzymes
Source: Aging (Albany NY). 2016 Dec 4;8(12):3375–88. doi: 10.18632/aging.101126 (PMC5270674; doi:10.18632/aging.101126)
Supplement: Supplementary file 4 [file aging-08-3375-s004.doc]

**Supplementary Table 3** (Related to Figure 3C). HNE modified proteins in senescent satellite cells.

| Protein spot no^a^ | Identified protein name | Swiss-Prot accession no^b^ | Mascot score^c^ | Sequence coverage (%)^d^ | No. of matched peptides^e^ | No. of sequenced peptides^f^ | Theoretical protein mass (Da)^g^ | Theoretical PI^h^ | RMI ratio^i^ |
| --- | --- | --- | --- | --- | --- | --- | --- | --- | --- |
| 1 | Alpha-enolase | ENOA | 1670 | 47 | 36 | 16 | 47,169 | 7.01 | h |
| 2 | Alpha-enolase | ENOA | 950 | 47 | 31 | 13 | 47,169 | 7.01 | 3,4 |
| 3 | 26S protease regulatory subunit 10B | PRS10 | 268 | 30 | 14 | 6 | 44,173 | 7.09 | 3,8 |
| 3 | Fructose-bisphosphate aldolase A | ALDOA | 227 | 27 | 14 | 4 | 39,420 | 8.3 | 3,8 |
| 4 | Annexin A2 | ANXA2 | 1820 | 53 | 37 | 19 | 38580 | 7,56 | h |
| 5 | Annexin A2 | ANXA2 | 1030 | 47 | 28 | 15 | 38580 | 7,56 | 3,5 |
| 6 | Phosphoglycerate mutase 1 | PGAM1 | 140 | 41 | 9 | 6 | 28,804 | 6,45 | 1,8 |
| 7 | Triosephosphate isomerase | TPIS | 1410 | 57 | 25 | 15 | 26,669 | 6,45 | 1,7 |
| 8 | Peroxiredoxin-6 | PRDX6 | 500 | 45 | 20 | 6 | 25,035 | 6 | h |

Spots of interest were identified by MS as described in Experimental procedures. Protein spots no (a) refer to numbered spots on Fig 3C. For each spot, different parameters clarifying protein identification by MS are indicated [accession number (b), mascot score (c), % sequence coverage (d), no. of matched peptides (e), no. of sequenced peptides (f), theoretical protein mass (g) and theoretical PI (h)]. RMI ratio (i) represents the Relative Modification Index Ratio and h means that the RMI ratio is higher than 5.
